# Inter-tester reproducibility and inter-method agreement of two variations of the Beighton test for determining Generalised Joint Hypermobility in primary school children

**DOI:** 10.1186/1471-2431-13-214

**Published:** 2013-12-21

**Authors:** Tina Junge, Eva Jespersen, Niels Wedderkopp, Birgit Juul-Kristensen

**Affiliations:** 1 Institute of Regional Health Services, University of Southern Denmark, Odense, Denmark; 2 Department of Physiotherapy, University College Lillebaelt, Odense, Denmark; 3 Institute of Sports Science and Clinical Biomechanics, University of Southern Denmark, Odense, Denmark; 4 Institute of Occupational Therapy, Physiotherapy and Radiography, Bergen University College, Bergen, Norway

**Keywords:** Hypermobility, Beighton tests, Reproducibility, Standardised protocol, Children

## Abstract

**Background:**

The assessment of Generalised Joint Hypermobility (GJH) is usually based on the Beighton tests, which consist of a series of nine tests. Possible methodological shortcomings can arise, as the tests do not include detailed descriptions of performance, interpretation nor classification of GJH. The purpose of this study was, among children aged 7-8 and 10-12 years, to evaluate: 1) the inter-tester reproducibility of the tests and criteria for classification of GJH for 2 variations of the Beighton test battery (Methods A and B) with a variation in starting positions and benchmarks between methods, and 2) the inter-method agreement for the two batteries.

**Methods:**

A standardised three-phase protocol for clinical reproducibility studies was followed including a training phase, an overall agreement phase and a study phase. The number of participants in the three phases was 10, 70 and 39 respectively. For the inter-method study a total of 103 children participated. Two testers judged each test battery. A score of ≥5 was set as the cut-off level for GJH. Cohen's kappa statistics and McNemar´s test were used to test for agreement and significant differences.

**Results:**

Kappa values for GJH (≥5) were 0.64 (Method A, prevalence 0.42) and 0.59 (Method B, prevalence 0.46), with no difference between testers in Method A (p = 0.45) and B (p = 0.29). Prevalence of GJH in the inter-method study was 31% (A) and 35% (B) with no difference between methods (p = 0.54).

**Conclusions:**

Inter-tester reproducibility of Methods A and B was moderate to substantial, when following a standardised study protocol. Both test batteries can be used in the same children population, as there was no difference in prevalence of GJH at cut point 5, when applying method A and B. However, both methods need to be tested for their predictive validity at higher cut-off levels, e.g. ≥6 and ≥7.

## Background

Generalised Joint Hypermobility (GJH) represents a variation of normal joint mobility, often defined as an increase in mean joint range of motion +2 SD [1]. Its prevalence among children varies from 4-40%, depending on age, gender, ethnicity, and the tests and criteria for classification used [2]. Joint hypermobility diminishes throughout childhood as a result of physiological changes in the connective tissue [2,3].

The assessment of GJH is usually based on tests using a dichotomous principle, such as the Beighton tests (BT) [4], rather than measurement of joint motion in degrees by goniometer on a continuous scale. The BT consists of nine tests, which seem to be reproducible in adults, as do the criteria for classification [5,6]. Two studies evaluating the BT and criteria for samples of children found the inter-tester reproducibility of the single tests in the BT to be moderate to almost perfect (κ 0.44-0.82) when performed by experts [7], while inter-tester reproducibility of criterion ≥6/9 was found to be substantial (κ 0.78) [8]. Those studies did not report if a standardised protocol for reproducibility studies was followed, leaving some uncertainty about the overall agreement and prevalence in the sample population. Information about prevalence is an important consideration before calculating and interpreting kappa, due to the problem that kappa values are influenced by prevalence well below or above 50% [9]. Using a method where an equal number of positive and negative tests are obtained – ‘the prevalence 0.50-method’, can be a purposeful sampling to obtain a pre-set prevalence. This method is feasible and solves one of the main drawbacks of using kappa statistics in reproducibility studies [6,9].

The lack of a standardised format for BT in published reproducibility studies, combined with a wide range of cut-off levels for GJH by different authors, makes comparison of the BT score problematic across studies and influences clinicians’ evaluation of the prevalence of GJH among children [10]. To facilitate and enhance scientific information exchange and fundamental discussions about GJH, the need for a standardised scientific protocol for future studies is obvious [9], especially when studying long-term consequences of GJH.

A methodological shortcoming is that the BT does not include detailed descriptions of the tests nor a definition of the criteria for classification of GJH. The BT was a modification of Carter and Wilkinson’s test for simply describing the population assessed in studies [4,11], rather than a diagnostic test. Consequently, none of the basic illustrations or descriptions of the BT state precisely how the tests should be performed, leaving researchers and clinicians to make their own choices regarding how to perform and interpret the tests. The BT seem inconsistent regarding the starting positions, performance, benchmarks and thereby the resultant outcome score. Different starting positions and benchmarks may affect the prevalence of GJH, influencing the validity of inter-study comparisons, and making the test of the predictive validity of BT in a cohort of children more difficult. To our knowledge, there are no studies comparing test batteries, where the single tests of BT are performed slightly different, yet still in accordance with the original test description.

The first purpose of this study was to determine the inter-tester reproducibility of tests and scoring criteria for two different test batteries for performing the BT (hereafter referred to as Method A and Method B) in a standardised protocol format. The second purpose was to determine the inter-method agreement of the prevalence of GJH of Methods A and B, using the criterion of a positive BT ≥5 for GJH.

## Methods

### Study design

#### Inter-tester reproducibility

**Figure 1:**
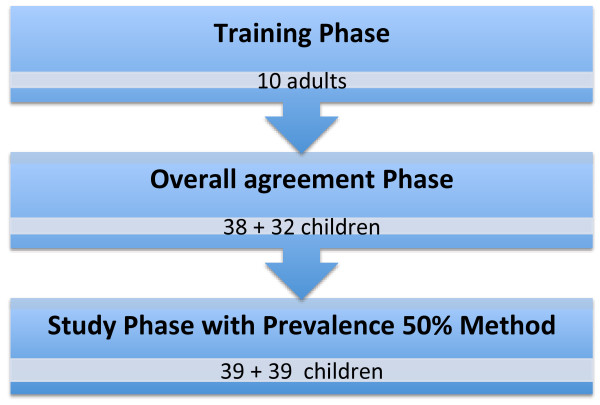
The inter-tester reproducibility study included a three-phase study with a training phase, an overall agreement phase and a test phase.

For the inter-tester reproducibility studies, a standardised protocol for clinical reproducibility studies was followed, including a three-phase study with a training phase, an overall agreement phase and a test phase [9] for each of the two different test batteries, Method A and Method B (Figure 1).

Phase 1 The training phase was performed in an open study in order to discuss and standardise every detail of performing and interpreting the BT among testers, thus improving their ability to follow strict test procedures, whether these were on adults or on children. In this phase, the testers were not blinded to GJH status or test results. The training phase was carried out in 10 adult cases (fellow physiotherapy students).

Phase 2 Using a blinded study, the main aim of the overall agreement phase was to obtain an overall percentage agreement of at least 80% for finding ≥5 positive tests out of 9 as the criterion for GJH. In this phase, testers were blinded with respect to both GJH status and the other testers results. Two observers were responsible for the randomisation of the test order, the selection of Method A or B and instructing the children not to comment on their status and the test outcome. A total of 38 children were included in Method A and 32 children in Method B, distributed by 57% boys and 43% girls with an average age of 7.4 years.

**Figure 2:**
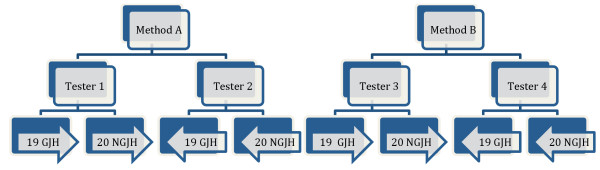
**Flow-chart for the 0.50 prevalence index method, study phase (Phase 3) for Methods A and B.** GJH: Generalised Joint Hypermobility, NGJH: Non-Generalised Joint Hypermobility.

Phase 3 In the test phase, the aim was to determine the kappa value (agreement adjusted by chance), using a blinded study, while ensuring an approximate 50% prevalence in order to optimise the kappa statistics validity [12,13]. Knowledge about the children with GJH score ≥5 found in Phase 2 was used to select children in advance for the test phase (Phase 3), so as to recruit as many children with GJH as possible. As a result, 19 children with GJH and 20 children without GJH from Method A and Method B, were sent to the allocated testers (Figure 2). The test phase consisted of 39 children, who were tested with both Methods A and B, and by all four testers. There were 54% boys and 46% girls with an average age of 9.6 years (Table 1).

**Table 1:** Participants of the inter-tester reproducibility and the inter-method study

**Inter-tester study**	**Phase 2**	**Phase 3**	**Inter-method study**	
**Age (min; max)**	7.4 (7;10)	9.6 (7;11)	**Age (min;max)**	8.7 (7-12)
**Sex (boys%)**	57	54	**Sex (boys%)**	52
**Total**	70	39	**Total**	103

#### Inter-method agreement

For the inter-method agreement study of the prevalence of GJH, the a priori choice of comparing data from Tester 1 with Tester 3, and Tester 2 with Tester 4, was arbitrarily used. The prevalence of GJH for both Methods A and B was compared with the criterion of ≥5/9 as a cut-off level.

The inter-method agreement study involved data from 103 consecutively recruited children, who had been tested in both Method A and Method B during the inter-tester reproducibility study. Six children were not a part of the inter-method analysis, as they due to lack of time were only tested with one method*.* All together, 62 children (60%) represented 7-8 year olds and 41 children (40%) 10-12 year olds (Figure 1).

#### Participants

Participants were healthy public school children from two different grades: first grade (7-8 years) and fourth grade (10-12 years).

Exclusion criteria were pain in the involved joints on the day of testing and movement restrictions, such as mild cerebral palsy, which would affect the results of the tests.

The grades are representing the youngest and oldest children in the CHAMPS Denmark part 1- The Childhood Health, Activity and Motor Performance School Study Denmark, a longitudinal cohort study of 1300 children in the Municipality of Svendborg [14,15]. The Committee on Biomedical Research Ethics for Southern Denmark approved the experimental protocol (jnr. S-20080047 HJD/csf). For this sub study of the CHAMPS Denmark part 1, The Regional Scientific Ethical Committee for Southern Denmark considered the experimental protocol as non-invasive. Therefore, the study was exempt from the obligation of ethical approval from the ethical committee. Parents of each participating child received written information according to the Declaration of Helsinki [16] and before examination each child gave oral consent to participate in the study. Parents were after consultation with the Regional Ethical committee of Southern Denmark asked to react if they did not want their child to participate.

### Methods

The two methods of BT were both in accordance with the original text of Beighton et al [4]. The original article from Beighton et al has a rather imprecise description of the tests, with no description of the procedures for each test. This is among others the reason, why there is so much diversion regarding the BT, and very few of these methods have been tested for reproducibility. The tests were performed with slightly different starting positions and benchmarks as this reflects daily clinical practice (Additional file 1). Besides variation in starting positions and benchmarks, the test batteries also differed in whether the tests were performed active or passive, how they were influenced by gravity and whether the surrounding soft tissue was in a stretched or relaxed position (Additional file 2). The current authors (TJ and EJ) made detailed descriptions regarding starting positions and benchmarks for the two different BT batteries (Additional file 2).

The BT started with a visual demonstration by the tester of the single test along with an oral instruction on how to perform the test before the children performed the test themselves. In the two methods, the children were asked to bring the joint to the most extreme position according to Methods A and B, tested consecutive by four different testers with approximately half an hour between testing sessions. All tests were performed in a random order with respect to right and left sides and to the test sequence.

A positive single test in the BT counted as 1 point, giving a maximum of 9 points, as previously described by Beighton et al [4]. A cut-off level for classification of GJH in children is internationally not established, as the predictive validity of GJH, for this time being, is not known. Due to the lack of predictive validity, an a priori cut-off level of ≥5/9 for GJH was chosen in the current study. Earlier studies have suggested different cut-off levels for classification of hypermobility in a child population: ≥4/9, ≥5/9 and ≥6/9 [8,17,18].

The same four testers evaluated the two different test batteries; two testers (Tester 1 and Tester 2) for Method A and two testers (Tester 3 and Tester 4) for Method B (Figure 2). The testers were physiotherapy students in the last year bachelor program, well trained in the performance and the interpretation of the BT.

#### Data analysis and statistics

For the inter-tester reproducibility studies of Method A and Method B, Cohen’s kappa statistics were used for each of the single tests and for the criterion for classification of GJH. Kappa values were classified as <0.0 = poor, 0.0-0.20 = slight, 0.21-0.40 = fair, 0.41-0.60 = moderate, 0.61-0.80 = substantial, and 0.81-1.00 = almost perfect [19].

McNemar’s test was used to test for significant differences between the two testers within each method, with *p* < 0.05 as the level of significance. For the inter-method study of comparing the prevalence obtained by method A and B, McNemar’s test was used to determine marginal homogeneity.

All calculations and statistical analyses were conducted in STATA (version 12.0) (Statacorp, College Station, Texas, USA).

## Results

In Phase 1, the tests for the knees and the elbows needed the most training and discussion and the test description was revised to gain final precision and equivalent interpretation.

In Phase 2, the overall agreement was 0.95 (Method A) and 0.81 (Method B) for the BT scoring criterion of ≥5/9. These agreements were deemed acceptable for continuing with Phase 3 for the inter-tester reproducibility of the tests and scoring criteria, in addition to the inter-method agreement for the criterion of GJH.

### Inter-tester reproducibility of tests and criteria

In Phase 3, kappa values varied from 0.49-0.94 (Method A) and from 0.30-0.84 (Method B) for the nine single tests in the batteries (Table 2). In 8 out of 9 tests, Method A had the highest agreement and the largest kappa value with a mean percentage agreement of 87%, while Method B had a mean percentage agreement of 81%. The mean kappa value for all tests was 0.70 (Method A) and 0.59 (Method B).

**Table 2:** Phase 3. Overall agreement, kappa values and prevalence (%) of Beighton tests and criteria in method A and method B

	**Overall agreement**	**Overall agreement**	**Kappa**	**Kappa**
			**Method A**	**Method B**
	**Method A**	**Method B**	**(Prevalence%)**	**(Prevalence%)**
**First finger, right**	97%	92%	0.94 (65%)	0.84 (63%)
**First finger, left**	95%	92%	0.89 (62%)	0.82 (68%)
**Fifth finger, right**	85%	77%	0.69 (46%)	0.43 (27%)
**Fifth finger, left**	74%	74%	0.49 (49%)	0.48 (41%)
**Elbow, right**	82%	80%	0.63 (42%)	0.60 (49%)
**Elbow, left**	87%	77%	0.73 (40%)	0.54 (47%)
**Knee, right**	85%	64%	0.62 (28%)	0.30 (46%)
**Knee, left**	85%	72%	0.62 (28%)	0.43 (42%)
**Forward bending**	95%	97%	0.64 (8%)	0.84 (9%)
**BT ≥5 GJH classification**	82%	80%	0.64 (42%)	0.59 (46%)

The body part with the highest agreement and kappa value was the first finger on the right hand for both Methods A and B (97%, κ 0.94 resp. 92%, κ 0.84) and the first finger on the left hand (95%, κ 0.89 resp. 92%, κ 0.82) (Table 2). The most difficult body parts to judge were the knees (mean 85%, κ 0.62 Method A, mean 68%, κ 0.37 Method B) and the elbows (mean 85%, κ 0.68 Method A, mean 79%, κ 0.57 Method B) (Table 2).

For the BT criteria for classification of GJH (≥5) in Phase 3, the prevalence was 42% (Method A), 46% (Method B) with kappa values moderate to substantial: 0.64 (Method A), 0.59 (Method B) (Table 3). There was no significant difference (McNemar’s Test) in the prevalence determined by testers within each method: p = 0.45 (Method A), p = 0.29 (Method B).

**Table 3:** Inter-method agreement presenting prevalence and kappa of Beighton score ≥4 and GJH classification by Beighton score ≥5

**Inter-method**	**Prevalence**		**McNemar significance probability**
	**Method A**	**Method B**	
**BT ≥5**	31%	35%	0.54
**GJH classification**			

### Inter-method agreement for the criterion of GJH

In the inter-method study, the prevalence of GJH when using the criterion of ≥5/9 was 31% (Method A) and 35% (Method B) with no difference between the methods when using McNemar´s Test (p = 0.54) (Table 3).

## Discussion

The inter-tester reproducibility of the test items of Methods A and B was moderate to substantial (κ 0.49-0.94 (mean 0.70) Method A, 0.30-0.84 (mean 0.59) Method B), using a standardised study protocol. The described methods for performing the BT are reproducible for children aged 7-8 and 10-12 years, using a cut-off level of ≥5/9 for classification of GJH. No significant difference in prevalence was found when using the two current test batteries.

Only two studies [7,8] have evaluated the inter-tester reproducibility of BT in a child population of a similar age, both with kappa values identical to the ones in the current study (0.69 (only four tests) [7], 0.78 [8] and 0.70 Method A [current study]).

The present kappa values were highest in tests that had the starting positions and simple benchmarks clearly described and easily identified, namely the test of the first finger and forward bending. The body part with the highest agreement and kappa was the first finger on the right hand for both Methods A and B (97%, κ 0.94 resp. 92, κ 0.84) and the first finger on the left hand (95%, κ 0.89 resp. 92, κ 0.82). The forward bending test had high overall agreement (95% resp. 97%) in the current study, but diverging kappa values from moderate to almost perfect kappa values (κ 0.64 resp. 0.84), affected by low prevalence. The findings were in accordance with a previous reproducibility study of GJH in children tested by trained physicians, who specialised in rheumatology, with kappa values of 0.82 for the first finger and 0.82 for forward bending [7]. In adults with GJH, the kappa value for the first finger was >0.94 [6].

The current most difficult body parts to evaluate were the knees, the elbows and the fifth fingers when visually estimating range of motion (ROM) in degrees (≥10° for knees and elbows and ≥90° for the fifth fingers). This was in accordance with the study by Hansen [7], with kappa values of 0.68 for the elbows and only 0.44 for the knees, judged by trained rheumatologists. However, that study did not include an overall percentage agreement phase, which may be the main reason for the poor reproducibility. In a previous study, reproducibility of tests for the elbows and the fifth fingers for adults was correspondingly low (κ <0.61), but for the knees kappa was as high as >0.85, possibly due to a prevalence close to 0.50 for the knees [6].

Comparing visual judgements with goniometer measurements represents a general challenge, but visual judgement is part of daily clinical practice. This problem was illustrated in a child study, where goniometry was used to measure the passive bilateral hyperextension of the knees along with visual judgements [20]. The children were placed into three sub-groups covering: the not hypermobile (BT score 0-4); the children with increased mobility (BT score 5-6); and the children being hypermobile (BT score 7-9). These three sub-groups were used for analysis of concurrent validity presenting significant differences between the exact degrees by goniometry and the total scores classified as the three sub-groups. The difference between BT scores 5-6 and 7-9 for knee extension was only 2 degrees, making an accurately visual judgement difficult. Also, the visual judgment of ROM in degrees for the single test was not validated against goniometry, potentially biasing the results, as the presence of hypermobile knee joints in the third sub-group could be low and therefore affect the mean ROM for knee extension.

Concurrent validity between goniometer measurements in degrees and visual judgment of the score of the single test was also evaluated in a pilot study, with, in contrast, no significant difference in the prevalence of GJH (criterion ≥6/9) in a child population, evaluated by goniometer measurements in degrees and visual judgment [21]. However, when comparing the individual tests, the prevalence for the five single tests was dissimilar for the elbows and especially the knee, judged by goniometer and visual estimates (right knee 2% resp. 18%, left knee 6% resp. 18%). This difference was obvious by both in-experienced and non-experienced physiotherapists [21]. The visual judgment of the shoulder position during evaluation of elbow hyperextension could also be a potential source of violation, as the angle of the elbow may seem dissimilar, if the shoulder is not placed in the starting position instructed.

The challenges of judging ROM visually and by goniometer was confirmed in a systematic review, where the reproducibility of knee extension, with or without test standardisation, varied from Kappa (PABAK) -0.02 (pre-standardisation of test) to 0.88 (post-standardisation of test) by rheumatologists [22,23]. In general, both goniometric measures and visually estimated measures were above ICC 0.59 for adults with or without diagnoses in the aforementioned systematic review including seven studies for knee extension measures [23].

In the current study, a higher mean kappa was seen for Method A (0.70) as for Method B (0.59) and with the largest kappa discrepancy for the right knee (A 0.62, B 0.30) and left knee (A 0.62, B 0.43). A possible explanation for this divergence could be familiarisation of Method A, as this method was used in another study carried out by the same testers. Alternatively, visual estimation of range of motion in degrees is challenging with the subject in a supine position. The differences in the two knee tests are the starting positions and the direction of gravity, as in Method A the child´s limb plus gravity affects the load on the knee, whereas in method B the tester applies a self-selected force to load the knee. This force may vary with the enthusiasm of the tester and the cooperation of the child [24]. In the study by Smits-Engelsman et al [20], the knee test was also performed in a supine position, while other studies have an upright starting position [4,6].

Other differences between the current study and the studies previously mentioned [4,6,7,20] involve dissimilar starting positions, such as testing the thumbs with the elbows extended [4,6,20] or flexed [7]. This dissimilarity might make a difference to the score, as the surrounding soft tissue will be tested in a stretched or a relaxed position. Other differences in starting positions may not have an impact on score, as in the test of the elbows with the arms in a shoulder abducted [6] versus flexed position [4,20].

We do not know whether the current results would be similar in a group with Hyper Mobility Syndrome (HMS), as the present study is a reproducibility study, where the aim is to test the reproducibility of only the BT in a normal and relevant population for our upcoming studies. A requirement of such study is to keep the testing conditions and the subject conditions as stable as possible for the test rounds. It could be anticipated that test results of BT in subjects with HMS would differ from first to second round due to increased pain, but this needs to be studied in a future study. Such considerations were bases for having pain as exclusion criteria in the present study.

Test differences and any resultant impact on scores complicate the interpretation and comparison of results across studies of GJH. This is the reason why consensus on a clear and unambiguous standard for test performances must be reached [25]. With standardised and detailed test protocols, increasing the agreement of the outcome scores, higher reproducibility values for the BT are likely to be attainable [9]. As the BT is a part of diagnostic criteria for conditions such as Marfan syndrome, EDS and HMS, the importance of clear, standardised protocols for making uniform clinical decisions is obvious.

Despite standardised test protocols, kappa values for reproducibility studies of tests for GJH are often not high, as the magnitude of kappa is affected by the prevalence of the condition in the population [26]. A practical method for independency of prevalence is to influence this in advance by ´the prevalence 0.50-method´ [9] as in the current study and the study by Juul-Kristensen et al [6]. For inter-tester reproducibility studies, both blinded testers will find an equal number of participants with positive and negative tests, whom will be tested by the other tester, and this way trying to get as close as possible to a prevalence of 0.50 [9].

Theoretically, kappa can also be adjusted for high or low prevalence, as well as bias, using PABAK (prevalence-adjusted bias-adjusted kappa) [26]. By subsequently calculating the average prevalence and bias (0.50) in the analysis, an indication of the likely effects of prevalence and bias is obtained. As the PABAK coefficient relates to a hypothetical situation in which no prevalence or bias effects are present, prevalence and bias must be presented in addition to the obtained value of kappa [26].

Both of these methods (0.50 method and PABAK) are ways of adjusting the prevalence, which can be an advantage when studying a condition found in only a small proportion of the population. The use of methods for adjusting prevalence may demonstrate a more reasonable evaluation of tests, provided the adjustment method is described.

The prevalence found in this reproducibility study was deceptively high (31% Method A, 35% Method B) using a cut-off level of ≥5/9, however this cut-off level was chosen with the 50%-prevalence method for purpose.

In European population studies, a prevalence of 16.8 – 46.4% has been found for the same age groups and the same cut-off level [8,17,20,27,28] depending on the way the BT was performed. In order to follow the cohort over time, determining the predictive validity for criteria, a higher cut-off level for classification of GJH is needed, as recommended by other authors [8,20].

The strength of this study was the high number of participating children in both the inter-tester and the inter-method study. To our knowledge, no studies have compared and evaluated 2 different ways of performing the BT batteries, although such differences are likely to occur in clinical practice. As in this study, small differences in the way the BT is performed may not have an impact on the prevalence when using a relatively low cut-off level, but at higher cut-off levels, slightly different starting positions and benchmarks may have a large influence on the prevalence. Consequently, standardised test protocols are recommended in order to attain high reproducibility for the single tests affecting the total BT score. This study took place in a school setting, and therefore, the prevalence of GJH is likely to be a realistic representation of that found in the general Danish child population.

## Conclusions

The inter-tester reproducibility of Methods A and B was moderate to substantial, when following a standardised study protocol. The described BT and criteria for classification of GJH are reproducible for children and therefore suitable for comparative studies of children, when using a GJH criterion of ≥5/9.

However, both methods need to be tested for their predictive validity at a higher cut-off level, e.g. ≥6 and ≥7.

## Abbreviations

GJH: (Generalised Joint Hypermobility); BT: (Beighton tests); ROM: (Range of Motion); PABAK: (prevalence-adjusted bias-adjusted kappa).

## Competing interests

The authors declare that they have no competing interests.

## Authors’ contributions

TJ, EJ and BJK contributed to the design of the study. TJ and EJ collected the data. TJ, EJ and BJK performed the data management. TJ, EJ and NW performed the data analysis and were in charge of data interpretation. TJ and EJ wrote the manuscript. All authors participated in data interpretation and contributed to manuscript revision. All authors read and approved the final version.

## Pre-publication history

The pre-publication history for this paper can be accessed here:

http://www.biomedcentral.com/1471-2431/13/214/prepub

## Supplementary Material

Additional file 1 Performance of the two BT batteries, Methods A and B, in accordance to the original text and description of starting position. Click here for file

Additional file 2 Test protocol for Beighton test and criteria for Generalised Joint Hypermobility as applied in Method A and Method B. Click here for file
