# Supplementary material for: Inter-tester reproducibility and inter-method agreement of two variations of the Beighton test for determining Generalised Joint Hypermobility in primary school children
Source: BMC Pediatr. 2013 Dec 21;13:214. doi: 10.1186/1471-2431-13-214 (PMC3878084; doi:10.1186/1471-2431-13-214)
Supplement: Additional file 1 — Performance of the two BT batteries, Methods A and B, in accordance to the original text and description of starting position. [file 1471-2431-13-214-S1.pdf]

	Original description of test - Beighton	Starting position Beighton	Starting position Method A	Starting position Method B
First finger	Passive apposition of the first finger to the flexor aspects of the forearm.	Shoulder flexed to 90°, elbow straight, dorsiflexion of the wrist.	Shoulder in neutral, elbow flexed, palmar flexion of the wrist.	Shoulder in neutral, elbow extended, palmar flexion of wrist, fingers pointing towards the floor
Fifth finger	Passive dorsiflexion of the little finger beyond 90°.	Straight wrist, flat hand resting at table. Position of elbow not illustrated.	Quadruped: Elbow extended, 90˚ angle between the forearm and the wrist, fingers extended.	Sitting with the forearm and hand resting on a table: Elbow in 90˚ flexion, straight wrist, fingers extended.
Elbow	Hyperextension of the elbow beyond 10°.	Shoulder flexed to 90°, upper arm in a horizontal angle, elbow straight, forearm supinated, hand loose.	Shoulder in 90˚ flexion, forearm/hand supinated.	Shoulder in 90˚ abduction, forearm/hand supinated.
Knee	Hyperextension of the knee beyond 10°.	Standing up, feet together, knee extended.	Standing up, feet almost together, knee extended.	Lying in supine. Heels resting on a box (20 cm), knee extended.
Trunk	Forward flexion of the trunk, with knees straight, so that the palms of the hands rest easily on the floor.	Standing up, feet together, knees straight, flexion of the hips and forward bending of the trunk.	Standing with the feet together: Forward flexion of the trunk with the knees extended and the palms of the hands touch the floor.	Standing with shoulder-width distance between feet: Forward flexion of the trunk with the knees extended and the palms of the hands touch the floor.

Appendix 1. Performance of the two BT batteries, Methods A and B, in accordance to the original text and description of starting position.
