# Supplementary material for: Inter-tester reproducibility and inter-method agreement of two variations of the Beighton test for determining Generalised Joint Hypermobility in primary school children
Source: BMC Pediatr. 2013 Dec 21;13:214. doi: 10.1186/1471-2431-13-214 (PMC3878084; doi:10.1186/1471-2431-13-214)
Supplement: Additional file 2 — Test protocol for Beighton test and criteria for Generalised Joint Hypermobility as applied in Method A and Method B. [file 1471-2431-13-214-S2.pdf]

Appendix 2.

Test protocol for Beighton test and criteria for Generalised Joint Hypermobility as applied in Method A and Method B.

Method A	Method B
**First finger: Apposition to forearm**<br>Standing: Passive apposition of the thumb to the flexor aspect of the forearm (elbow flexed, wrist in volar flexion). Tested on right and left side.<br>The tester demonstrates the test and asks: ”Can you make your thumb touch the forearm?”. Positive score = 1 point for each side reaching a benchmark of the thumb touching the forearm.<br>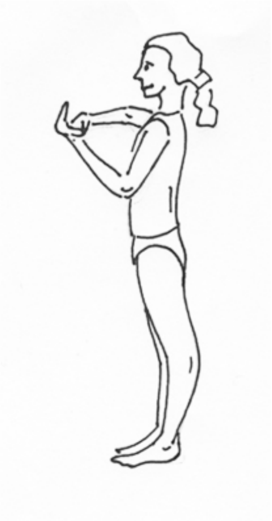	**First finger: Apposition to the forearm**<br>Standing: Passive apposition of the thumb to the flexor aspect of the forearm (90° shoulderflexion, elbow extended and wrist in volar flexion). Tested on right and left side.<br>The tester demonstrates the test and asks: ”Can you make your thumb touch the forearm?”. Positive score = 1 point for each side reaching a benchmark of the thumb touching the forearm.<br>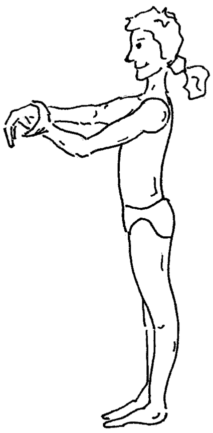
**Fifth finger: Extension ≥90°**<br>Quadruped: Passive dorsiflexion of the fifth finger ≥90˚ (elbows extended, 90˚ angle between the forearm and the wrist, fingers extended). Tested on right and left side. The tester demonstrates the test and asks: ”How far can you bend your little finger back?”. Positive score = 1 point for each side reaching a benchmark ≥90°.	**Fifth finger: Extension ≥90°**<br>Sitting with forearm and hand resting on a table: Passive dorsiflexion of the fifth finger ≥90˚(elbow in 90˚ flexion, fingers extended). Tested on right and left side. The tester demonstrates the test and asks: ”How far can you bend your little finger back?”. Positive score = 1 point for each side reaching a benchmark ≥90°.

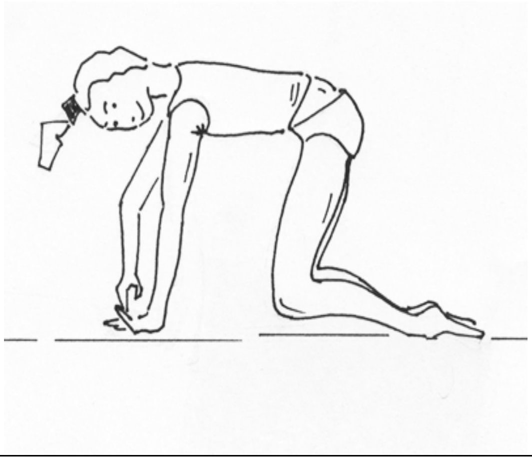	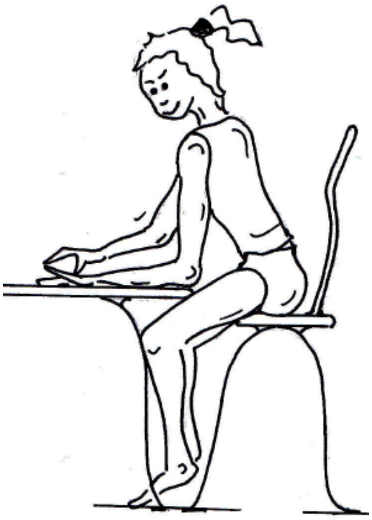
**Elbow: Hyperextension ≥10°**<br>Standing: Passive hyperextension of the elbows ≥10˚ (shoulder flexion 90˚, forearm/hand supinated). Tested on right and left side.<br>The tester demonstrates the test and asks: ”How much are you able to stretch out your elbows?”. Positive score = 1 point for each side reaching a benchmark ≥10˚.	**Elbow: Hyperextension ≥10°**<br>Standing: Passive hyperextension of the elbows ≥10˚. (shoulder abduction 90˚ and forearm/hand supinated). Tested on right and left side.<br>The tester demonstrates the test and asks: ”How much are you able to stretch out your elbows?”. Positive score = 1 point for each side reaching a benchmark ≥ 10˚.
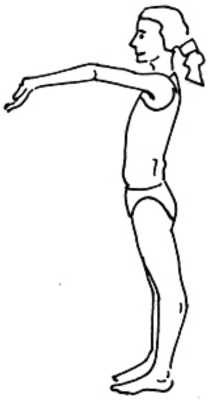	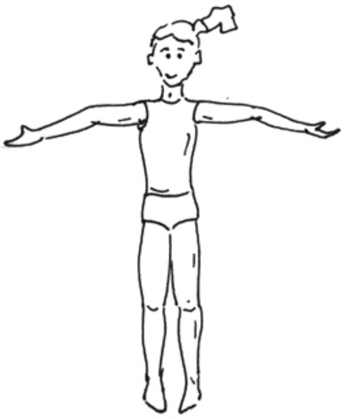
**Knee: Hyperextension ≥10°**<br>Standing: Hyperextension of the knees ≥10˚. Tested on right and left side.<br>The tester demonstrates the test and asks: ”How much are you able to stretch out your knees?”. Positive score = 1 point for each side reaching a benchmark ≥10˚.	**Knee: Hyperextension ≥10°**<br>Testperson in supine. Heels resting on a box (20 cm): Hyperextension of knees ≥10˚. Tested on right and left side.<br>The tester demonstrates the test and asks: ”How much are you able to stretch out your knees?”. Positive score = 1 point for each side reaching a benchmark ≥10˚.

<table>
<tr>
<td>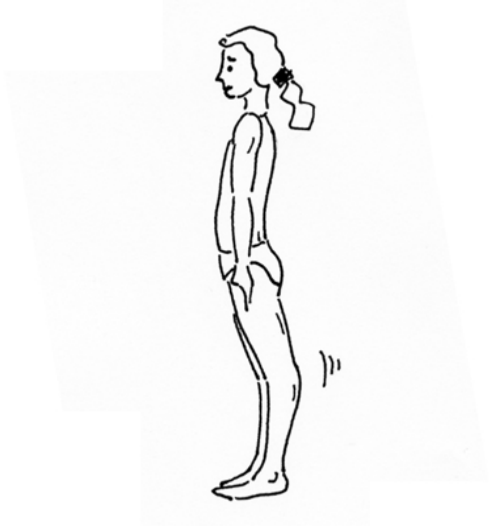</td>
<td>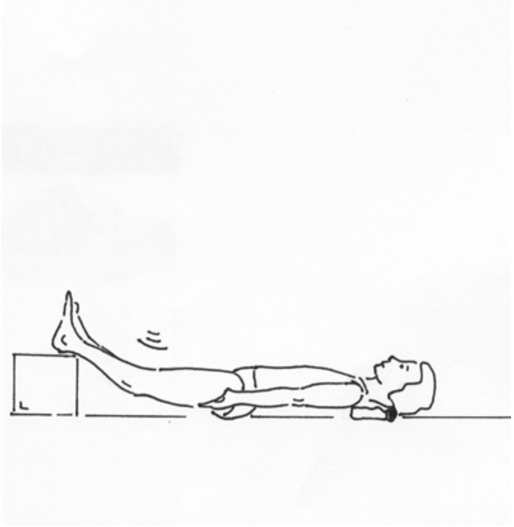</td>
</tr>
<tr>
<td>Forward bending: Palms at floor
Standing with the feet together: Forward flexion of the trunk with the knees extended and the palms of the hands touching the floor. The tester demonstrates the test and asks: ”Can you, keeping the knees straight, touch the floor with your hands?”. Positive score = 1 point for reaching a benchmark of touching the floor with the palms of the hands and the knees extended.
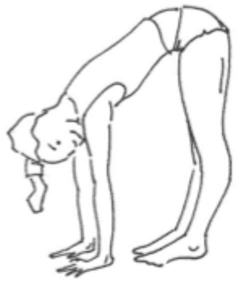</td>
<td>Forward bending: Palms at floor
Standing with shoulderwidth distance between feet: Forward flexion of the trunk with the knees extended and the palms of the hands touching the floor.
The tester demonstrates the test and asks: ”Can you, keeping the knees straight, touch the floor with your hands?”. Positive score = 1 point for reaching a benchmark of touching the floor with the palms of the hands and the knees extended.
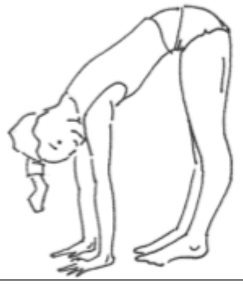</td>
</tr>
</table>
